# Elimination of *bla*_KPC−2_-mediated carbapenem resistance in *Escherichia coli* by CRISPR-Cas9 system

**DOI:** 10.1186/s12866-023-03058-7

**Published:** 2023-10-26

**Authors:** Shuan Tao, Huimin Chen, Na Li, Yewei Fang, He Zhang, Yao Xu, Luyan Chen, Wei Liang

**Affiliations:** 1 Department of Clinical Laboratory, The First Affiliated Hospital of Ningbo University, Ningbo, China; 2 School of Medicine, Jiangsu University, Zhenjiang, China; 3 Bengbu Medical College, Bengbu, China; 4 School of Medicine, Ningbo University, Ningbo, China; 5 Department of Blood Transfusion, The First Affiliated Hospital of Ningbo University, Ningbo, China

**Keywords:** CRISPR-Cas9, *Klebsiella pneumoniae*, Antimicrobial resistance, *bla*_KPC−2_

## Abstract

**Objective:**

The purpose of this study is to re-sensitive bacteria to carbapenemases and reduce the transmission of the *bla*_KPC−2_ gene by curing the *bla*_KPC−2_-harboring plasmid of carbapenem-resistant using the CRISPR-Cas9 system.

**Methods:**

The single guide RNA (sgRNA) specifically targeted to the *bla*_KPC−2_ gene was designed and cloned into plasmid pCas9. The recombinant plasmid pCas9-sgRNA(*bla*_KPC−2_) was transformed into *Escherichia coli (E.coli)* carrying pET24-*bla*_KPC−2_. The elimination efficiency in strains was evaluated by polymerase chain reaction (PCR) and quantitative real-time PCR (qPCR). Susceptibility testing was performed by broth microdilution assay and by E-test strips (bioMérieux, France) to detect changes in bacterial drug resistance phenotype after drug resistance plasmid clearance.

**Results:**

In the present study, we constructed a specific prokaryotic CRISPR-Cas9 system plasmid targeting cleavage of the *bla*_KPC−2_ gene. PCR and qPCR results indicated that prokaryotic CRISPR-Cas9 plasmid transforming drug-resistant bacteria can efficiently clear *bla*_KPC−2_-harboring plasmids. In addition, the drug susceptibility test results showed that the bacterial resistance to imipenem was significantly reduced and allowed the resistant model bacteria to restore susceptibility to antibiotics after the *bla*_KPC−2_-containing drug-resistant plasmid was specifically cleaved by the CRISPR-Cas system.

**Conclusion:**

In conclusion, our study demonstrated that the one plasmid-mediated CRISPR-Cas9 system can be used as a novel tool to remove resistance plasmids and re-sensitize the recipient bacteria to antibiotics. This strategy provided a great potential to counteract the ever-worsening spread of the *bla*_KPC−2_ gene among bacterial pathogens and laid the foundation for subsequent research using the CRISPR-Cas9 system as adjuvant antibiotic therapy.

**Supplementary Information:**

The online version contains supplementary material available at 10.1186/s12866-023-03058-7.

## Introduction

The emergence and rapid spread of multidrug-resistant pathogenic bacteria pose great challenges to global public health and clinical medicine [1, 2]. Horizontal gene transfer (HGT) mediated by mobile genetic elements (MGEs) can promote the exchange of genetic material between different bacteria and is an important reason for the development of multi-drug resistance in bacteria [3, 4]. Investigation results from clinical medicine and ecological environment have shown that horizontal transfer of antibiotic resistance genes (ARGs) (especially plasmid-mediated horizontal transfer of resistance genes) promotes the spread of multi-drug resistance across regions and populations [5]. Carbapenems are one of the most effective antimicrobial agents against infections caused by multiple-resistant Gram-negative bacilli (GNB) [6]. Unfortunately, carbapenem-resistant *Enterobacteriaceae* (CRE) disseminated worldwide and exacerbated the failure in clinical treatment [7]. Of particular concern is the occurrence and prevalence of *Klebsiella pneumoniae* carbapenemase(KPC)-producing *K. pneumoniae* (KPC-*Kp*) [8]. *K. pneumoniae* resistance to carbapenem is mainly associated with the acquisition of carbapenem-producing hydrolysis β-lactamase, and KPC-2 is one of the most commonly identified in *K. pneumoniae* in China [9]. Carbapenemase genes tend to be located on conjugative plasmids and mobile genetic elements that can horizontally spread within as well as between species [10]. Therefore, it is necessary to discover and develop novel antimicrobial strategies to combat the wide dissemination of carbapenemase genes *bla*_KPC−2_ and limit the spread of plasmid-borne resistance. The CRISPR-Cas9 system can specifically recognize and target DNA sequences carrying antibiotic-resistance genes and limit the spread of drug resistance genes, which has potential applications in the prevention and control of the spread of bacterial drug resistance [11, 12].

Clustered regularly interspaced short palindromic repeat (CRISPR)-Cas systems are adaptive immune system in bacteria and archaea to defend against the HGT mediated by MGEs and has been applied in genome editing [13]. The type II CRISPR/Cas9 system is widely used for target-specific genome engineering. The endonuclease Cas9, guided by a sgRNA, recognizes and cleaves double-stranded DNA (dsDNA) and causes double-stranded breaks at the target site near the protospacer adjacent motif (PAM) sequence, followed by non-homologous end joining (NHEJ) or homologous recombination (HR) [14]. Due to the lack of an efficient NHEJ pathway in most prokaryotes, Cas9-mediated DSB cannot be repaired spontaneously, thereby achieving the purpose of editing the target gene [15]. Thus, the CRISPR-Cas9 system can be used to combat contagious infections and develop novel antimicrobial drugs [16].

In the present study, we investigated the effect of the CRISPR-Cas9 system on the elimination of *bla*_KPC−2_ gene and explored the application potential of the CRISPR-Cas9 system to control the spread of *bla*_KPC−2_ resistance. The results demonstrated that the prokaryotic CRISPR-Cas9 system can eliminate the *bla*_KPC−2_ plasmid in drug-resistant model bacteria and re-sensitive to carbapenemase by removing the resistant plasmids, which offered a novel strategy to combat the dissemination of antibiotic resistance genes among bacterial pathogens at the molecular level and maximize the advantages of CRISPR technology in the field of anti-bacterial resistance.

## Materials and methods

### Bacterial strains, plasmids, and growth conditions

Bacterial strains and plasmids used or constructed in this study are listed in Table 1. *E. coli* DH5α and *E. coli* BL21 were used as competent cells for the plasmid transformation experiments and propagation. The *bla*_KPC−2_ genes were obtained from plasmid pHS10842 isolated and stocked in this laboratory. The pCas9 plasmid carrying the Cas9 endonuclease and sgRNA-binding site and tracRNA (Addgene, plasmid number 42,876) was obtained from Hunan Fenghui Biotechnology Co., Ltd [17]. *E. coli* was grown in Luria-Bertani (LB, 5 g yeast extract, 5 g NaCl, and 10 g tryptone per litre) broth or on LB agar (LB supplemented with 15 g agar per litre) plates at 37 °C. For plasmid maintenance, 100 µg/ml of ampicillin (Amp) and chloramphenicol (50 mg/L) were added when necessary.

**Table 1:** Bacterial strains and plasmids used in this study

Bacterial strains and plasmids		Reference or source
Bacterial strains *E*. *coli* DH5α *E. coli* BL21	F-,φ80dlacZΔM15, Δ(lacZYA-argF)U169, deoR, recA1, endA1, hsdR17(rk-, mk+), phoA, supE44, λ-, thi-1, gyrA96, relA1 F- ompT hsdSB(rB- mB-) gal dcm (DE3)	This study
Plasmids pHS10842 pCas9 (Addgene,42,876) pCas9-sgRNA pET24 pET24-*bla*_KPC−2_	*bla*_KPC−2_-harboring plasmids Cm^r^, tracRNA, gRNA, and cas9 expression plasmid; Cm^r^, pCas9 cloned with sgRNA targeting *bla*_KPC−2_; Kan^r^, expression vector Kan^r^, recombinant vector derivative with *bla*_KPC−2_ gene	Laboratory stock [15] This study Laboratory stock This study

Notes: *E. coli*, *Escherichia coli*; Kan^r^, kanamycin-resistant; Cm^r^, chloramphenicol-resistant

### Plasmid construction

The plasmids were constructed using standard molecular biology techniques [18]. The *bla*_KPC−2_ gene sequence was amplified from the pHS10842 plasmid by PCR using a forward primer containing the EcoRI restriction enzyme site (5’-GAATTCCGGGGCGAAGGTTAAATGGG-3’, the underline indicates EcoRI site)

### Transformation experiments

The competent cells including *E.coli* BL21 containing pET24-*bla*_KPC−2_ were prepared and used for transformation assay following the protocol [19]. The chemical transformation was performed by mixing 100µL of chemically competent cells with 10µL of plasmid pCas9-sgRNA incubated on ice for 30 min, followed by a heat shock for the 90s at 42 °C and finally incubated on ice for 2 min. Then transformed cells were grown in 1ml LB media and incubated at 37 °C, with 200 rpm vigorous shaking for 1 h. The transformants were selected on LB agar containing chloramphenicol (50 mg/L) and cultured at 37 °C overnight. The empty plasmid pCas9 was used as the negative control.

### Evaluation of the CRISPR-Cas elimination efficiency

#### PCR detection of *bla*_KPC−2_ plasmid clearance efficiency

After transformation, 20 single clones were randomly selected to evaluate the elimination efficiency. The CRISPR plasmid was detected by PCR with primers pCas9-F/R(pCas9-F:CGGCGTTATCACTGTATTGCACGG and pCas9-R: TGTGTACGCGATGGATACCG). At the same time, the colonies were screened for target gene deletions by PCR with primers *bla*_KPC−2_-F/R (*bla*_KPC−2_-F: GTTCCGGTTTTGTCTCCGAC and *bla*_KPC−2_-R: GCCGTGCAATACAGTGATAA). The strain carrying the *bla*_KPC−2_ gene was used as a positive control.

#### E-test antimicrobial test strips detect bacterial resistance phenotype

Antimicrobial sensitivity assays were performed using E-test strips (bioMérieux, Sweden) according to standard operating procedures on colonies with negative PCR product bands and positive control colonies. The minimum inhibitory concentration(MIC) of imipenem (IPM) (carbapenem resistance) was obtained by E-test strips according to the guidelines of the Institute for Clinical and Laboratory Standards (2017) [20].

#### Quantitative PCR (qPCR) detection of *bla*_KPC−2_ copy number changes

To further analyze the efficiency of pCas9-sgRNA in eliminating the *bla*_KPC−2_ gene, the genomic DNA of experimental and control groups was extracted using the TIANamp Bacteria DNA Kit (Tiangen, Beijing, China). And the change of *bla*_KPC−2_ copies number at 2,4,8,16, and 32th hour after transformation into the CRISPR-Cas9 system were calculated using the SYBR Green fluorescent dye of fluorescent quantitative PCR (qPCR) with primers specific for *bla*_KPC−2_ gene (KPC-F: TGTGCTTGTCATCCTTGTT and *bla*_KPC−2_-R: GAACCTGCGGAGTGTATG) and using the 16 S gene (16 S-F: AGAGTTTGATCCTGGCTCAG and 16 S-R: CTGCTGCCTCCCGTAGGAGT) as the internal reference. All reactions were run in triplicate. Relative gene expression of *bla*_KPC−2_ copy number in the experimental group compared to the control group was calculated by the 2^−ΔΔCT^ CT method.

### Statistical analysis

Statistical analysis was performed with GraphPad Prism version 9.0 (GraphPad Software Inc., San Diego, CA, USA). P < 0.05 was considered as statistical significance.

## Results

### Construction of the CRISPR-Cas9 plasmid and recombinant plasmid targeting the *bla*_KPC−2_ gene

According to the NGG PAM sequence (protospacer adjacent motif sequence) principle, the sgRNAs were designed for *bla*_KPC−2_ in the promoter region or near the ATG sequence in the targeted gene and were right after an NGG sequence. Then the oligonucleotide with 20nt was cloned into the pCas9 plasmid and obtained pCas9-sgRNA specifically targeting *bla*_KPC−2_. In addition, we selected the plasmid pET-24 as the backbone to construct a recombinant plasmid pET24- *bla*_KPC−2_ containing the *bla*_KPC−2_ gene.

### Validation of the effect of CRISPR-Cas9 cutting *bla*_KPC−2_ plasmid

**Fig. 1:**
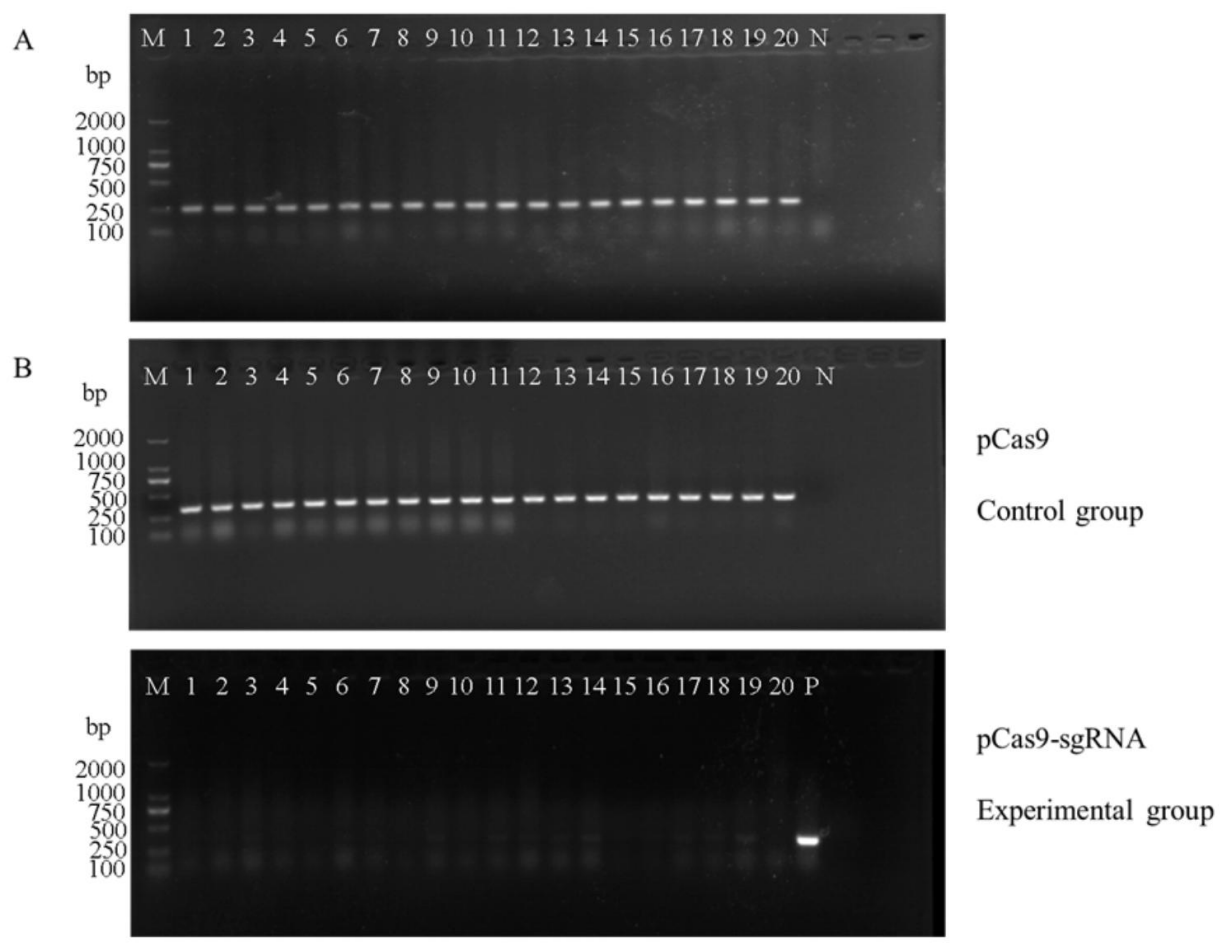
(**A**) Confirmation of cas9 gene presence in *E. coli* BL21 + pET24-*bla*_KPC−2_ by PCR amplification with primer pCas9-F/R. Lane M represents 2000 bp DNA molecular markers. The 1–20 lanes mean *E.coli* BL21 + pET24-KPC strain transformed with pCas9-sgRNA, lane N is a negative control. (**B**) Confirmation of *bla*_KPC−2_ gene elimination in BL21 + pET24-*bla*_KPC−2_ by PCR amplification with primer *bla*_KPC−2_ -F/R in the control group and experimental group (Note:Integration gel images presented in the main figures are cropped for clarity, full un-cropped gel images are presented in Supplementary Fig.)

To assess the efficiency of the CRISPR-Cas9 system in mediating plasmid elimination in *E. coli*. 20 single colonies were selected from the plates of the experimental group transformed with pCas9-sgRNA to *E.coli* BL21-pET24- *bla*_KPC−2_ and the plates of the control group transformed with the pCas9 empty vector, respectively. The results showed that all 20 colonies in the experimental group were negative for *bla*_KPC−2_, indicating that the presence of the *bla*_KPC−2_ gene failed to be amplified by PCR after specific cleavage by Cas9 nuclease. The shear success rate was 100% in the selected 20 transformants. In contrast, the Cas9 nuclease in the control group lacked the guidance of sgRNA and showed no specific cleavage of *bla*_KPC−2_. See Fig. 1. The PCR results indicate that the specific CRISPR system could cut and destroy the *bla*_KPC−2_ gene.

### Changes in bacterial phenotypes

**Fig. 2:**
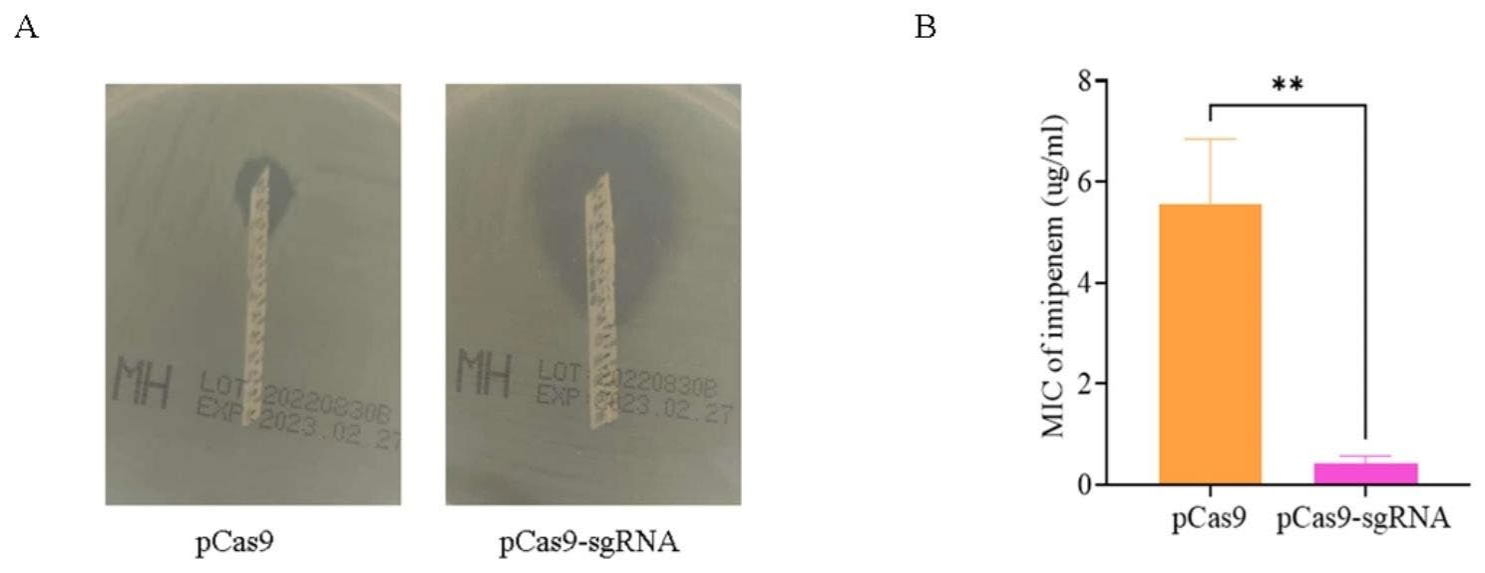
(**A**) Epsilometer test strips (Etest) to detect the MIC value of imipenem in the control group (pCas9) and experimental group (pCas9-sgRNA) (**B**) Statistical analysis of imipenem MIC value

The greatest threat to the clinical treatment of *bla*_KPC−2_-producing bacteria is their resistance to carbapenem antibiotics. E-test strips were used to detect the changes in the MIC value of imipenem in the experimental group. The results found that the bacterial resistance to imipenem was significantly reduced (n = 3, unpaired t-test *p* = 0.0025 after *bla*_KPC−2_ was specifically cleaved by the CRISPR system. The MIC value of the control group was 5.55 ± 1.30 µg/mL, while the MIC value of the experimental group dropped to 0.42 ± 0.15 µg/mL). See Fig. 2.

### Analysis of CRISPR system editing efficiency of *bla*_KPC−2_

**Fig. 3:**
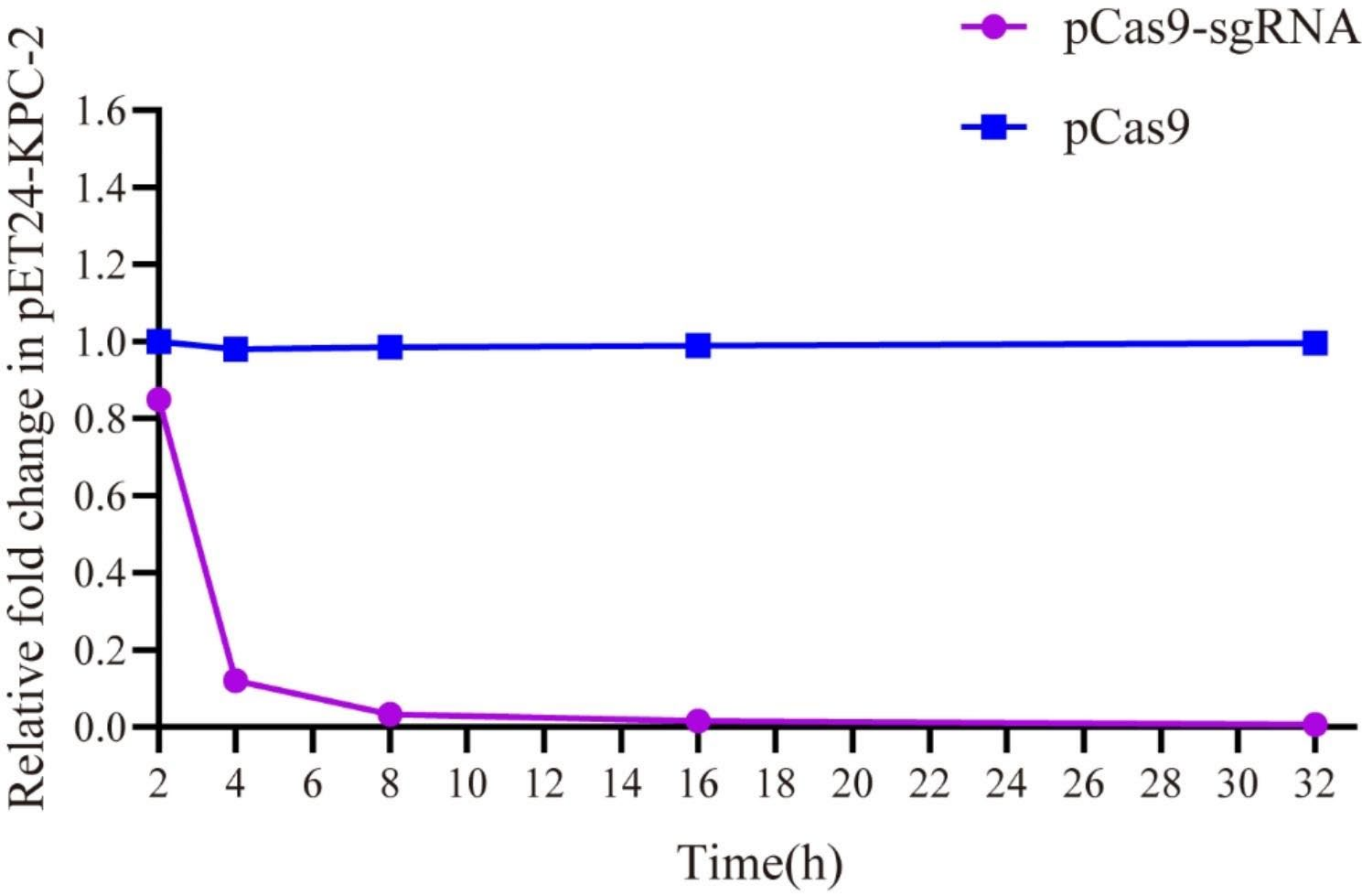
The relative copy number of plasmid pET24-*bla*_KPC−2_ at each time point

In this study, Quantitative PCR (qPCR) analysis of the efficiency of pCas9-sgRNA in clearing *bla*_KPC−2_ was performed to calculate the relative copy number of plasmids at each time point in the pCas9-sgRNA experimental group and the pCas9 control group after transformation of the CRISPR-Cas 9 system. See Fig. 3. The quantitative analysis found that there were significant differences in plasmid copy number between the experimental and control groups. The experimental group cleared about 80% of the resistant plasmid at the 4th hour and greater than 98.8% at the 8th hour, and the clearance of the resistant plasmid continued until the 32th hour. The results showed that the CRISPR-Cas9 system could effectively eliminate the *bla*_KPC−2_ gene. Although the *bla*_KPC−2_ plasmid has the potential to restore its copy, this experiment confirmed that no rebound in the copy amount of the drug-resistant plasmid was observed during the process of CRISPR clearing *bla*_KPC−2_.

## Discussion

Carbapenem-resistant *Klebsiella pneumoniae* (CR-*Kp*) has spread worldwide and brought severe challenges to the anti-infection treatment [21]. KPCs are the major factor causing the widespread of drug resistance in *K. pneumoniae* [22]. The wide spread of KPC is often closely associated with the horizontal gene transfer of mobile elements [23]. And plasmid-mediated acquired drug resistance is an important factor in promoting MDR-*KP* (Multidrug-Resistant *Klebsiella pneumoniae*) formation [24]. Therefore, the destruction and elimination of drug-resistant plasmids are crucial for the prevention and control of MDR-*KP* formation. The CRISPR-Cas system has the characteristics of specifically targeting drug resistance genes or plasmids and has attracted much attention as a potential strategy to prevent and control the formation of drug-resistant bacteria. CRISPR-Cas system is an adaptive immune system that prevents the HGT mediated by mobile genetic elements [25]. The CRISPR-Cas9 system has been widely used for gene editing in eukaryotes [26]. In recent years, the CRISPR-Cas9 system has been widely applied to the prevention and control of bacterial drug resistance [11, 27].

In this study, we applied the CRISPR-Cas9 system to antibiotic-resistant bacteria to eliminate *bla*_KPC−2_-carrying plasmid by designing sgRNA to target the resistant gene *bla*_KPC−2_ and verified that specific elimination of the resistant plasmid allowed resistant model bacteria to restore susceptibility to antibiotics. We quantitatively analyzed the clearance efficiency of CRISPR on drug-resistant plasmids and observed that the natural model drug-resistant plasmids achieved high clearance efficiency, showing the great application potential of the CRISPR system in eliminating bacterial drug resistance.

The CRISPR-Cas system can specifically identify and target the cleavage of genetic elements carrying drug-resistant genes or their transcripts and has application prospects for preventing and controlling bacterial multi-drug resistance. However, the off-target characteristics of the CRISPR-Cas system have always been a bottleneck for this method in biomedicine and clinical applications [28]. Combining experimental and analytical methods to design appropriate sgRNA for the target DNA sequence is a crucial first step to avoid off-target [29]. Studies related to CRISPR gene editing have reported that good sgRNA design can optimize the shearing efficiency of the CRISPR system [30, 31]. Especially in eukaryotic systems, different target sites can lead to differences in CRISPR shear efficiency, as well as different degrees of off-target effects [32, 33]. It has been indicated that the sgRNA sequence of different lengths may affect the curing efficiency of the plasmid. Zhang et al. [34] showed that knockout sgRNA with the potency of 17nt or 20nt varied in different host stem cells and this difference could be explained by the differential levels of Cas9 expression in different cell types. However, it has also been shown that the knockdown efficiency of 17nt sgRNA in human cells is similar to that of 20nt sgRNA, but its off-target mutagenic effect is greatly reduced. This may indicate that the effect of sgRNA sequence length varies from host to host [35].

The CRISPR-Cas gene editing system has been successfully applied to combating bacteria, virus, and genetic disorders. Wan et al [36] revealed that the CRISPR-Cas9 system can resist the conjugation process of the conjugation process of the IncI2 plasmid pHNSHP45 containing the colistin (*mcr-1*) resistance gene to the recipient bacteria. Integrating the CRISPR-Cas9 system targeting the erythromycin resistance gene on the pheromone response plasmid can effectively eliminate the erythromycin resistance gene of multi-drug resistant *E. faecalis* in the mouse intestine [37]. The DNA fragment of CRISPR-Cas9 carried by the phage capsid can be modified to eliminate only pathogenic bacteria or drug-resistant plasmids in mixed bacterial populations without affecting other populations, thereby achieving the purpose of selectively regulating bacterial populations [38, 39]. For example, using phage as a vector, sgRNA and Cas9 designed based on the conserved sequences of β-lactamase mutants were introduced into the target strain, successfully achieving the loss of activity of more than 200 β-lactamase gene mutants in pathogenic bacteria, making drug-resistant bacteria restoration of sensitivity to beta-lactam antibiotics [40]. However, the existence of extensive multidrug resistance (MDR) may be unsatisfactory to eliminate using a single non-essential target. Therefore, the efficiency of the CRISPR-Cas system can be improved by designing a CRISPR-Cas system to target the essential genes on the resistance plasmid or using a CRISPR array to establish multiple cleavage sites simultaneously. Studies have shown that two sgRNAs on a single structure can be used to target and remove *bla*_NDM−1_ and *bla*_CTX−M−15_ genes [41], which is important for reducing the spread of MDR. Rodriguez et al [37] used the CRISPR-Cas9 system to target the *tet(M)* and *erm(B)* genes conferring resistance to tetracycline and erythromycin, respectively and successfully reduced the drug resistance of *Enterobacter faecalis* in vitro and in vivo. Hao et al [42]. developed the plasmid curing system pCasCure based on the CRISPR-Cas9 system to precisely cut and define carbapenemase genes such as *bla*_NDM_, *bla*_KPC_, *bla*_OXA−48_ CRE and targeted prevalence of repA, repBp, and parApKpQIL plasmids to remove plasmids carrying carbapenemase resistance genes and re-sensitive CRE carbapenem antibiotics. The MIC value was reduced by more than 8 times. Scientists are attempting to develop a CRISPR-Cas9 system to restore antibiotic susceptibility of extended-spectrum-lactamase (ESBL)-producing *E. coli* by identifying a conserved target sequence among > 1000 ESBL mutants [43]. Therefore, combining experimental and analytical methods, designing the best sgRNA can improve the activity and specificity of sgRNA.

In addition, the efficacy of CRISPR-Cas9 mainly depends on the delivery efficiency of the system [44]. And since the transformation is a non-spontaneous mode, this form may be limited in clinical application. Therefore, other delivery methods such as plasmid conjugation, and phage vectors have been used in in vitro or in vivo models [38, 45]. Previous studies have found that it can be overcome by using nanomaterials as non-viral vectors to deliver the CRISPR-Cas9 system [46]. A variety of innovative polymers, lipids, and gold nanoparticles have been developed [47]. However, the integration of materials with CRISPR systems is still in its early stages. Based on plasmids, phages, nanoparticles, and other carriers to deliver CRISPR-Cas system-related biological macromolecules (nucleic acids or protein and nucleic acids) to bacteria, which is not only difficult to import but also the incoming biological macromolecules are easy to degradation by bacterial intracellular proteases or nucleases. Therefore, there are still great challenges in applying these technologies to medical clinics, livestock and poultry breeding, or ecological environments. The focus of the subsequent research is on how to efficiently present the CRISPR-Cas9 system to the bacterial cells. The endogenous CRISPR-Cas system is ubiquitous in bacteria. The use of small molecule compounds or physicochemical factors to activate the bacterial endogenous CRISPR-Cas system may provide new thinking directions for preventing and controlling bacterial multi-drug resistance caused by drug-resistant gene transfer. Clarifying the regulatory mechanism of the CRISPR-Cas system and identifying relevant regulatory target proteins is the basis and prerequisite for carrying out the above work. The successful application of the CRISPR-Cas system in the treatment of bacterial infections and the control of the spread of drug-resistant bacteria requires further research.

## Conclusion

In this study, we constructed specific prokaryotic CRISPR-Cas9 plasmid targeting the *bla*_KPC−2_ gene, demonstrated the ability of the CRISPR-Cas 9 system to eliminate *bla*_KPC−2_ plasmids carried in *E. coli*, and re-sensitize resistant strains to carbapenems, and showed the efficient clearance of resistant plasmids by the CRISPR system. In conclusion, the strategy of the CRISPR system targeting bacterial drug resistance plasmids can not only help kill drug-resistant bacteria, but also destroy the plasmid vector of drug resistance genes, thereby blocking the horizontal transfer of drug resistance genes, which provides a reference for the active prevention and control of bacterial drug resistance, and further research is needed to optimize drug administration methods to improve the efficiency of clinical application.

### Electronic supplementary material

Below is the link to the electronic supplementary material.

Supplementary Material 1

## Data Availability

The datasets used and analysed during the current study are available from the corresponding author on reasonable request.
