# Supplementary material for: Elimination of blaKPC−2-mediated carbapenem resistance in Escherichia coli by CRISPR-Cas9 system
Source: BMC Microbiol. 2023 Oct 26;23:310. doi: 10.1186/s12866-023-03058-7 (PMC10601263; doi:10.1186/s12866-023-03058-7)
Supplement: Supplementary file 1 — Supplementary Material 1 [file 12866_2023_3058_MOESM1_ESM.pdf]

Supplementary Fig information

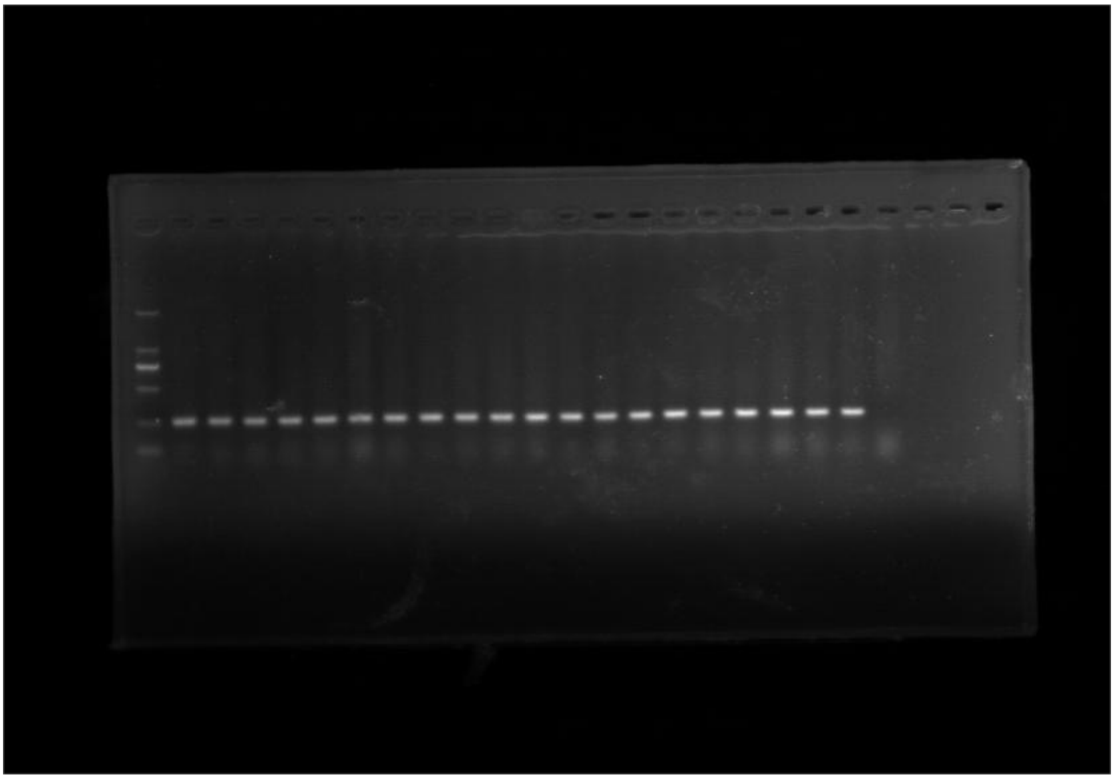

(A)Confirmation of cas9 gene presence in *E. coli* BL21+pET24-KPC by PCR amplification with primer pCas9-F/R.

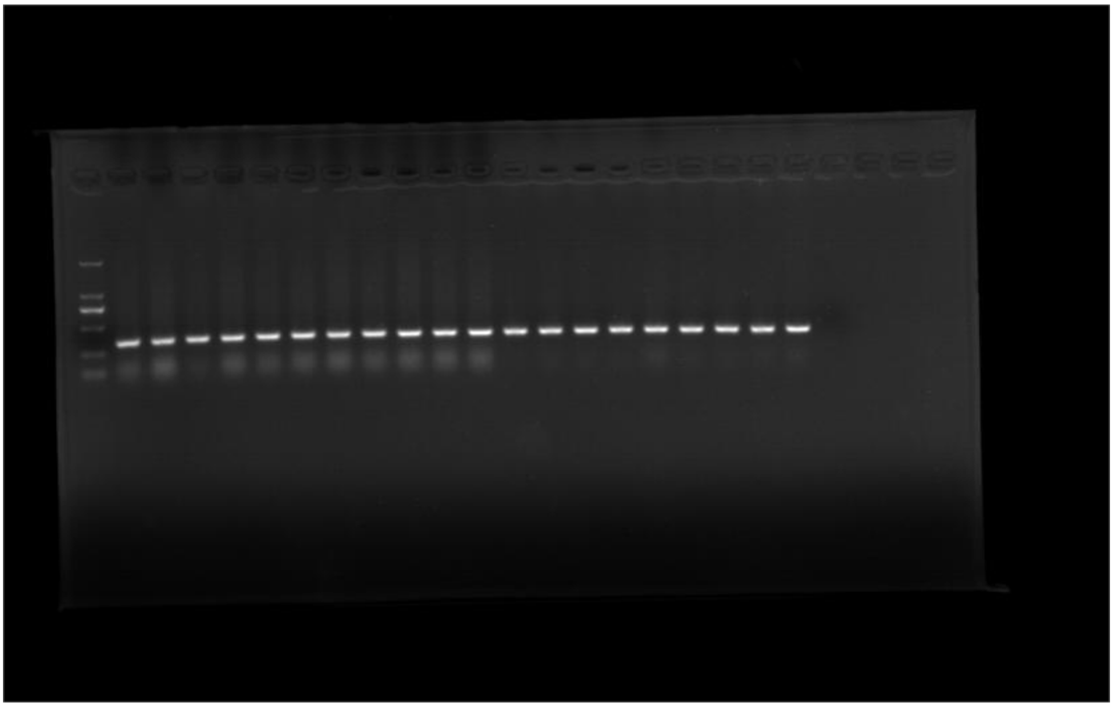

Confirmation of *blaKPC-2* gene elimination in BL21+pET24-KPC by PCR amplification with primer KPC-2-F/R in the control group.

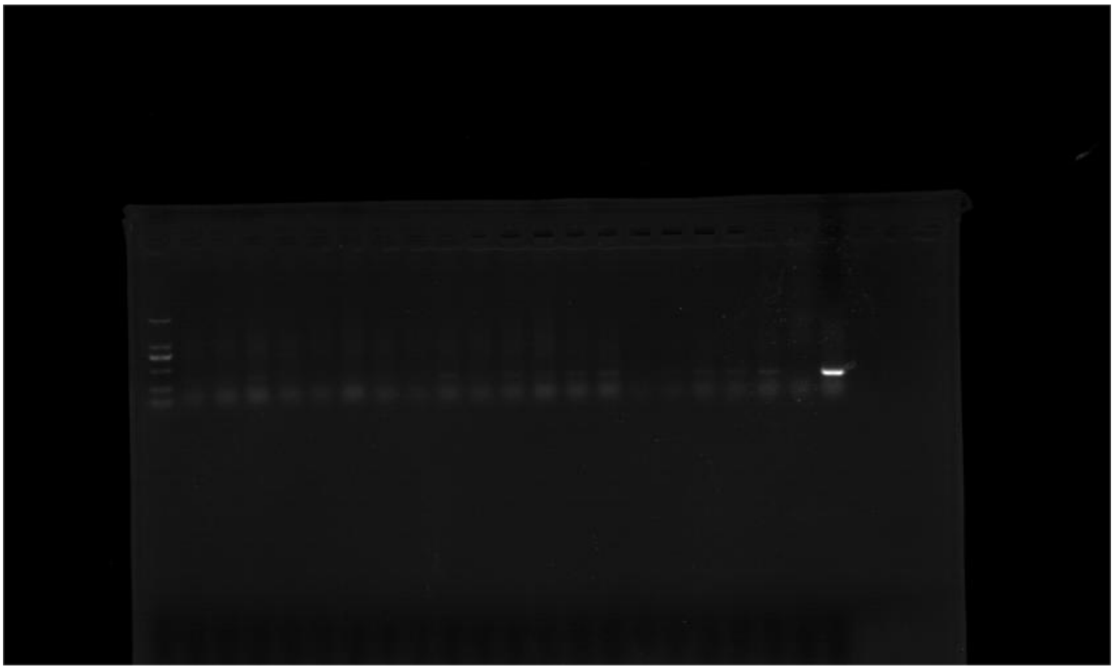

Confirmation of *blaKPC-2* gene elimination in BL21+pET24-KPC by PCR amplification with primer KPC-2-F/R in the experimental group.
